# Role of Vitamin D in the Hygiene Hypothesis: The Interplay between Vitamin D, Vitamin D Receptors, Gut Microbiota, and Immune Response

**DOI:** 10.3389/fimmu.2016.00627

**Published:** 2016-12-23

**Authors:** Allison Clark, Núria Mach

**Affiliations:** ^1^Health Science Department, International Graduate Institute of the Open University of Catalonia (UOC), Barcelona, Spain; ^2^Animal Genetics and Integrative Biology Unit (GABI), INRA, AgroParisTech, Université Paris-Saclay, Jouy-en-Josas, France

**Keywords:** vitamin D, vitamin D deficiency, vitamin D receptor, gut microbiota, hygiene hypothesis, autoimmune diseases, Western lifestyle

## Abstract

The hygiene hypothesis postulates that higher levels of cleanliness and improper exposure to microorganisms early in childhood could disturb the intestinal microbiome resulting in abnormal immune responses. Recently, more attention has been put on how a lack of sun exposure and consequently vitamin D deficiency could lead to less immune tolerance and aberrant immune responses. Moreover, vitamin D receptor (VDR) function has been positioned to be a critical aspect of immune response and gut homeostasis. Therefore, this review focuses on the role that the interaction between vitamin D, VDR function, and gut microbiome might have on autoimmune diseases in the context of the hygiene hypothesis. Literature shows that there is a high correlation between vitamin D deficiency, VDR dysfunction, gut microbiota composition, and autoimmune diseases. The biologically active form of vitamin D, 1,25(OH)_2_D_3_, serves as the primary ligand for VDRs, which have been shown to play a fundamental role in reducing autoimmune disease symptoms. Although the biological functions of VDR, the effects of its genetic variants, and the effects of epigenetic profiles in its promoter region are largely unknown in humans, studies in murine models are increasingly demonstrating that VDRs play a crucial role in attenuating autoimmune disease symptoms by regulating autophagy and the production of antimicrobial peptides, such cathelicidin and β-defensin, which are responsible for modifying the intestinal microbiota to a healthier composition. Remarkably, evidence shows that hormonal compounds and byproducts of the microbiota such as secondary bile acids might also activate VDR. Therefore, understanding the interaction between VDR and gut microbiota is of the utmost importance toward understanding the rise in autoimmune diseases in Western countries. We have gained insights on how the VDR functions affects inflammation, autophagy, and microbiota composition that could lead to the development of pathogenesis of autoimmune diseases, while confirming the role vitamin D and VDRs have in the context of hygiene hypothesis.

## Introduction

Strachan developed the hygiene hypothesis in 1989 with the notion that viral infections early in life and family size affect the risk of suffering from hay fever and allergic rhinitis (1). Since then, it has been suggested that the increased cleanliness and subsequent lack of proper microbial exposure in early childhood, a common occurrence in Western society, can disrupt the human microbiome causing a lack of indigenous microbes and aberrant immune responses (2–4).

An international systematic review on vitamin D, the gut microbiome, and the hygiene hypothesis (5) suggested that limiting the hygiene hypothesis to excessive cleanliness and early life infections does not fully explain the rise in autoimmune diseases or the susceptibility to asthma in Western countries. They postulated that less sun exposure and thus vitamin D deficiency as well as reduced exposure to ancient and beneficial microorganisms that “train” our immune systems from the uterus to postnatal life are inversely associated with immune tolerance and gut microbiota diversity (5). The critical role of vitamin D on the gut microbiome and developing fetal lung and immune system has been reviewed by the same authors (6); however, beyond vitamin D deficiency, we noted that the relationship between vitamin D receptor (VDR) functions, gut microbiota, and the rise in autoimmune diseases had not yet been extensively explored. With specific focus on the interaction between vitamin D, VDRs, gut microbiota, and immune responses, this review will allow greater understanding of how this interaction could lead to the development of an autoimmune disease. We hypothesize that the increasing rates of vitamin D deficiency coupled with VDR dysfunction and a lack of gut microbiota diversity are the key drivers of the rise in autoimmune diseases in Western countries.

## Materials and Methods

We conducted a systematic review and synthesis of relevant qualitative research according to the requirements established in the preferred reporting items for systematic review and meta-analysis protocols (7). The protocol was registered *a priori* with PROSPERO on April 11, 2016 (CRD42016037431).

### Eligibility Criteria and Literature Search Strategy

A systematic and comprehensive search of electronic databases, including MEDLINE, Scopus, http://ClinicalTrials.gov, the PROSPERO International Prospective Register of Systematic Reviews, Science Direct, Springer Link, and EMBASE was done from March 2016 to September 2016.

The search process was completed using the keywords: “hygiene hypothesis,” “gut microbiota,” “microbiota heritability,” “vitamin D,” “VDR,” “autoimmune disease,” and “immune tolerance.” The search was not restricted to the type of study (i.e., species, meta-analysis, case–control, prospective cohort studies, and reviews), sample size, year of publication, publication status, or follow-up. However, we only consulted articles published in English. Bibliographies of the identified reviews and original research publications were hand selected for additional studies that may have been missed by the database searches. All articles were exported to the reference database Zotero. Due to the nature of this review, no request was performed for the ethics committee’s approval.

### Data Extraction and Synthesis

Full copies of citations coded as potentially relevant were obtained, and those meeting the inclusion criteria were read in detail and data were extracted. One reviewer (Allison Clark) extracted information about the study aim, population and sample size, experimental design and duration of follow-up, species, individual characteristics, changes in the gut microbiota composition, and immune response and association or not with an autoimmune disease. The primary outcome was the gut microbiota profile, aberrant changes in the immune response, vitamin D status, VDR functions, or other clinically relevant outcomes related to autoimmune and immune-related conditions. Details were then checked by a second reviewer (Núria Mach). If eligibility could be determined, the full article was retrieved.

The articles and extracted data were read and the findings were organized into the following categories: (i) hygiene hypothesis, the gut microbiota, and the immune system; (ii) experimental articles about the possible relationship between disturbances of the gut microbiota and/or vitamin D_3_ deficiency, VDR dysfunction, and autoimmune diseases.

### Data Synthesis

A search conducted in March 2016 resulted in the following list of key terms combinations (hygiene hypothesis, the gut microbiota, and autoimmune disease = 5; vitamin D and autoimmune disease = 18; vitamin D3, VDR function, intestinal microbiota, and autoimmune diseases = 16). A total of 47 experimental studies and 54 reviews met the inclusion criteria and were included in the review. Most of the articles were reviews or randomized controlled trials. Periods of data collection spanned from 1989 to 2016, proving data from humans and animals models (i.e., mice and rats).

## Discussion

### Hygienic Western Lifestyle and Its Effects on the Gut Microbiome

Most studies about the hygiene hypothesis have focused on the depletion of indigenous microbiome diversity in the modern world and the rise of autoimmune disease prevalence (8). The human microbiome is the “forgotten organ” and is as unique as a fingerprint (9). Humans are home to a complex ecosystem of trillions of microbes such as archaea, small eukaryotes, fungi, parasites, viruses, and yeast (10). The gut microbiota is essential for host immune function, nutrient digestion, short chain fatty acids (SCFAs) production, vitamin synthesis, energy metabolism, intestinal permeability, protection from pathogens, and determining the host’s susceptibility to gastrointestinal infections (11, 12).

Commensal microorganisms, pathogens, and nutrients that pass through the intestinal lumen are the first point of contact with the enteric immune system, which plays a critical role in innate and adaptive immunological functions (13, 14). A constant cross talk occurs between intestinal epithelial cells, gut microbiota, and the gut-associated lymphoid tissue, which is mainly composed of Peyer’s patches, lymphoid nodules embedded in the submucosa of the small intestine, and lymphocytes distributed throughout the lamina propria (15). Epithelial cells, dendritic cells (DCs) located in Peyer’s patches and macrophages within the lamina propria present pattern recognition receptors such as toll-like receptor and nucleotide-binding oligomerization domain 2 (Nod2) receptors, which are responsible for different immune responses when facing dysbiosis or abiotic stress (16). Therefore, the gut microbiota is believed to be crucial for proper host immune development and response (17) and plays a key role in building up the host’s tolerance to foreign antigens (5).

The shift from Paleolithic times to industrialization has greatly affected the human microbiome, which is believed to be due to certain hygienic practices (18, 19). According to the review conducted by Rook (19), a hygienic lifestyle and cleanliness can generally be defined as an abuse of antibiotic and dewormer treatments that can decrease immune tolerance, antibacterial soaps and cleaners, drinking chlorinated water, and delayed exposure to viruses among newborns coupled with an excessive time spent indoors. All these practices can deplete indigenous microorganisms or “Old Friends” that help regulate the immune system (19). A hygienic lifestyle can also lead to decreased exposure to indigenous viruses such as Hepatitis A, pathogenic bacteria such as *Heliobacter pylori, Salmonella* spp., *Mycobacterium tuberculosis*, and parasites like helminths and *Toxoplasma gondii* (19). Anecdotal evidence has shown that parasitic infection diminishes or eliminates allergic reactions (11) probably because helminths can modulate the gut microbiota and DCs toward a more tolerogenic phenotype (20). For example, Nod2 knockout (KO) mice showed that intestinal helminth infection prevented the colonization of inflammatory *Bacteriodes vulgatus* and promoted the colonization of protective microbiota enriched in Clostridiales, which was caused by a T helper cell type 2 immune response (21). On the other hand, dewormer treatment decreased Clostridiales and increased Bacteroidales (21).

Studies comparing the fecal microbiota of indigenous populations vs. Westerners have shown that an overly hygienic lifestyle leads to less microbial diversity of the gut microbiota (22). In a landmark study of the Yanomami indigenous group who live in a rural area of the Amazon, Clemente et al. (23) discovered that these people who do not have an excessively hygienic lifestyle, spend hours outside, and do not take antibiotics presented 50 times more gut microbiome diversity than Americans and also suffered less autoimmune diseases. Additionally, indigenous diets tend to be much higher in dietary fiber, which can lead to a healthier gut microbiota composition that is lower in Firmicutes and higher in Bacteroidetes and the anti-inflammatory microbiota byproducts SCFAs (24). Dietary changes can account for up to 57% of gut microbiota changes, whereas the human genome accounts for no more than 12% (25), which could explain why Westerners have less microbial diversity in the gut given that the Western diet is characteristically low in fiber which can lead to less microbiota diversity (26, 27).

**Figure 1:**
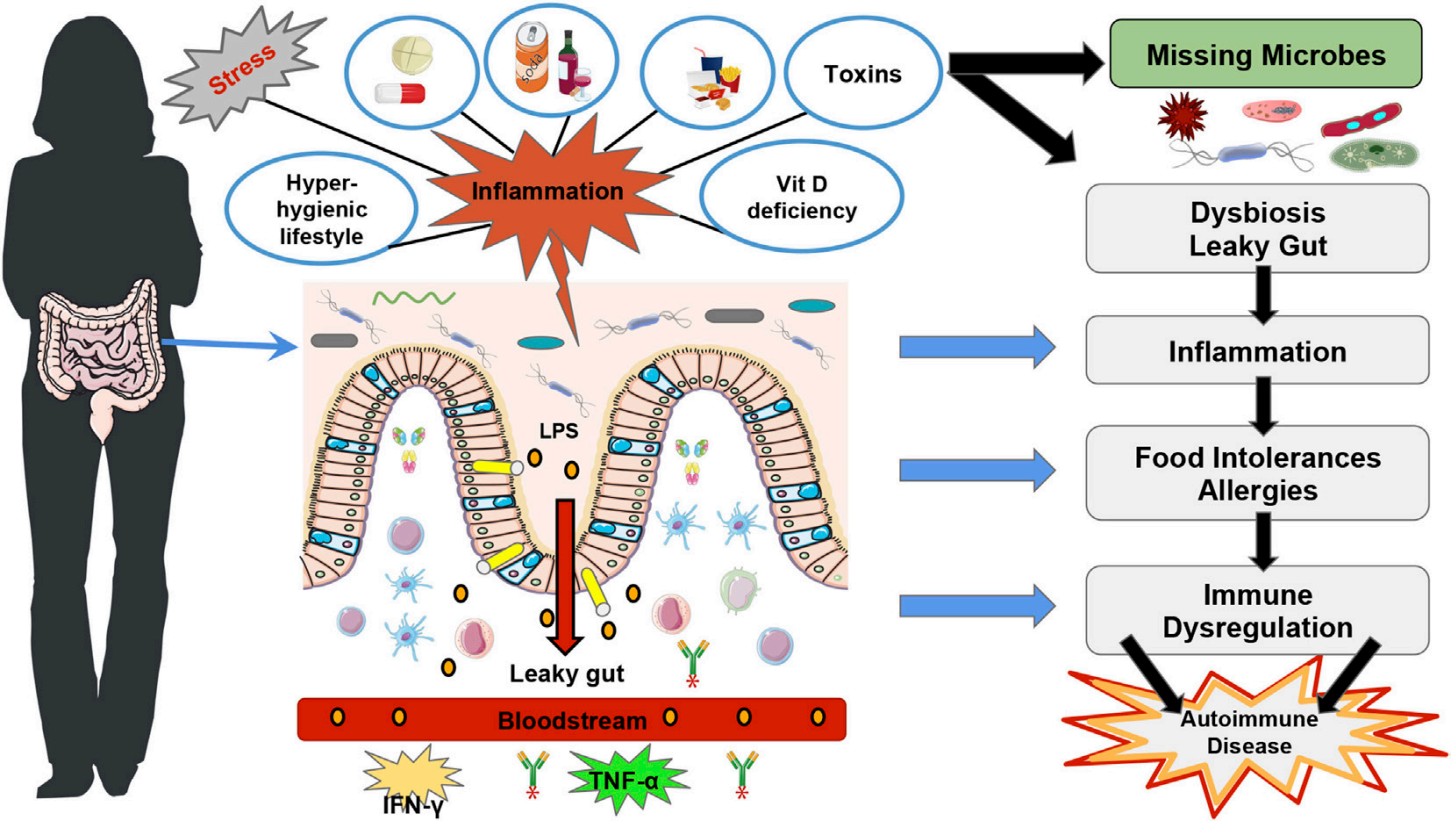
**Western lifestyle factors that lead to autoimmunity diseases**. The figure represents the main factors that contribute to the disruption of gut homeostasis and rise in autoimmune diseases in Western countries, such as stress, a hyper-hygienic lifestyle that includes deworming treatments at an early age, drinking chlorinated water (19), and excessive antibiotic treatments (34), the Western diet which is characteristically low in fiber and high in saturated fat (35), toxins (36), drugs (36–38), and vitamin D deficiency (39). Furthermore, Vitamin D deficiency and vitamin D receptor function have also been shown to disrupt gut homeostasis and consequently immune tolerance (40, 41). All of these factors can lead to intestinal dysbiosis, more susceptibility to pathogenic infections and intestinal permeability, which predispose to lipopolysaccharides translocation and trigger inflammatory immune responses such as TNF-α and IFN-γ (42). The loss of immune homeostasis can lead to food intolerances and allergies, which can subsequently lead to autoimmune disease development (19).

In summary, literature shows that a hygienic Western lifestyle can reduce gut microbial diversity (28) leading to “over zealous” immune responses, which could explain the increase in autoimmune diseases (Figure 1). However, autoimmune diseases, which are characterized by a loss of self-antigen tolerance (29) and increased auto-antibodies and/or auto-reactive lymphocytes (30), depend not only on the gut microbiota diversity and function but also on other factors such as vitamin D deficiency (5, 31) and VDR functions to regulate immune responses [Figure 1; (32, 33)].

### Vitamin D Deficiency, the Immune System, and Gut Microbiota

As reviewed by Litonjua and Weiss (6), vitamin D not only helps regulate calcium levels, blood pressure, and electrolytes, it is also an essential component of our immune system. Vitamin D deficiency is a contributing factor to the increasing rates of autoimmune diseases such as rheumatoid arthritis, systematic lupus erythematosus, multiple sclerosis (MS), type I diabetes, irritable bowel disease (IBD), and other autoimmune diseases (43).

**Figure 2:**
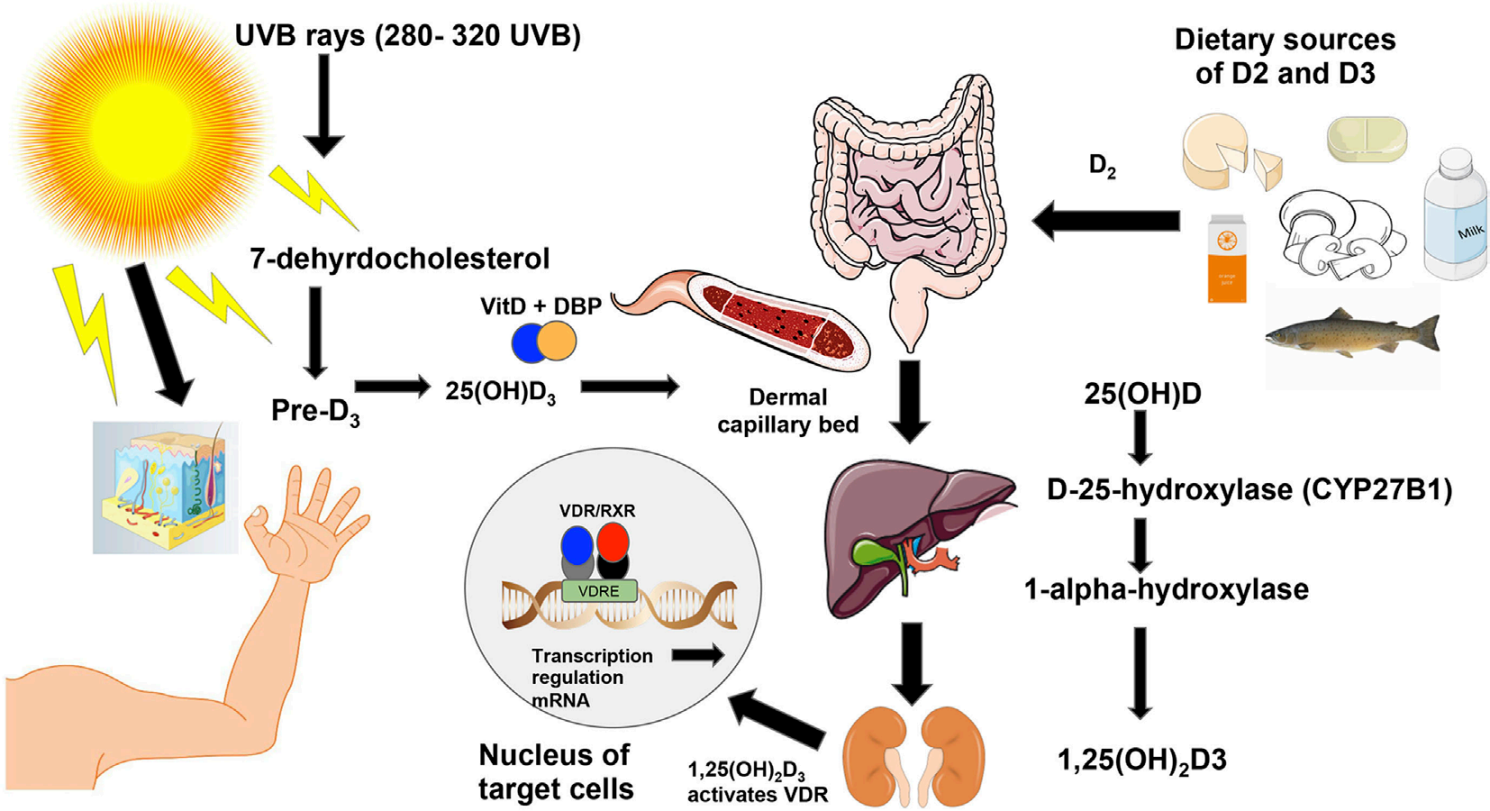
**Vitamin D_3_ synthesis from sun exposure and food**. Vitamin D is synthesized from sun exposure or consumption of foods rich in vitamin D. The ultraviolet B rays from the sun at frequencies between 280 and 320 hit the skin where 7-dehydrocholesterol is converted into pre-vitamin D_3_ and is then isomerized into vitamin D_3_ or cholecalciferol (46). Vitamin D-binding protein then facilitates vitamin D_3_’s entrance into the dermal capillary bed (39). On the right of the figure, ingested vitamin D_2_ (ergocalciferol) from food or supplements is incorporated into chylomicrons, which enter the lymphatic system and blood. Once in the bloodstream, both vitamin D_2_ and vitamin D_3_ move to the liver where the enzyme cytochrome P450 hydroxylates both forms of the vitamin to 25(OH)D_3_. Then, the CYP27B1 further hydroxylates 25(OH)D_3_ into the active form of the vitamin [1,25(OH)_2_D_3_] in the kidneys (39). Unlike D_2_ or other vitamin D metabolites, vitamin D_3_ is the active form of vitamin D that binds to the vitamin D receptor (VDR), which is located in about 30 different tissues (47). The activated VDR binds to vitamin D responsive elements (VDREs) to form a heterodimer (48), which can regulate directly or indirectly the expression of ~3% of the mouse and human genomes (49).

Western society’s lifestyle has led to people spending more time indoors and thus having less sun exposure which is believed to be a major cause of vitamin D deficiency (44). While diet can provide up to 10–20% of the human body’s requirements for vitamin D, ~90% of all needed vitamin D has to be photosynthesized in the skin through ultraviolet B rays [UVB; Figure 2; (45)]. The rays hit the skin converting 7-dehyrocholesterol into pre-vitamin D_3_, which is then isomerized into cholecalciferol or D_3_ (46). For this reason, vitamin D synthesis from solar rays can be affected by latitude, air pollution, season, time of the day, sunscreen use, and skin pigmentation (39). Vitamin D-binding protein binds to D_3_, which reaches the dermal capillary bed where it gets transferred from the bloodstream to the liver (39). On the other hand, ingested vitamin D_2_ or ergocalciferol passes through the small intestines and binds to chylomicrons, which enter the lymphatic system, and then bloodstream where they are transferred to the liver. In the liver, both vitamin D_2_ and vitamin D_3_, are hydroxylated by the enzyme cytochrome P450 to 25-hydroxyvitamin D_3_ (25(OH)D_3_). Then, the 25(OH)D_3_ is further converted to 1α,25-dihydroxyvitamin D_3_ [1,25(OH)_2_D_3_], the hormonally active secosteroid, by the 1-α-hydroxylase enzyme cytochrome P450 family 27 subfamily B member 1 (CYP27B1), primarily in the kidneys (39). Finally, 1,25(OH)_2_D_3_ binds to the VDR, which is located in about 30 different tissues (47) and can regulate the expression of more than 1,000 genes in the genome (Figure 2). For further details on the biological functions of VDR, see section “Discussion.”

Generally, the ingestion of 1,000 IU vitamin D_2_/day increases the 1,25(OH)_2_D_3_ levels ~10 ng/mL, though individual results may vary (31). An adult exposed to 1 minimal erythemal dose (slight pinkness to the skin 24 h after exposure) is equivalent to an oral intake of 20,000 IU (500 µg) of vitamin D_2_ (31). Whereas arm and leg exposure of 0.5 erythemal dose is equivalent of oral intake of 3,000 IU of vitamin D_2_ (39). About 20 min of sun exposure on the arms and face between latitudes 42°N and 42°S is equivalent to 200–400 IU of vitamin D_3_ ingestion (50).

Besides the sun, exposure to endocrine disrupting chemicals such as bisphenol A and phlalates, which are widely used industrial compounds found in several commercial products, may alter serum 25(OH)D_3_, which is the metabolite of vitamin D used to measure vitamin D levels in adults. These chemicals have been found to modify the expression of cytochrome P450 and CYP27B1 genes in mice (51). Therefore, exposure to common chemicals found in Western society may also be a contributing factor to the rise in vitamin D deficiency.

Approximately one billion people worldwide suffer from vitamin D deficiency (47), which is generally defined as <20 ng/mL (50 nmol/L) (52). An estimated 20–80% of the population in the Canadian and European population is vitamin D deficient while approximately one-third of the U.S. population is deficient (39), yet there is a surprising lack of research in Vitamin D deficiency in African and South American populations (53). The populations most at risk for suffering a deficiency are infants and children >5 years old, people 65 years and older, pregnant women (39), those with dark-skin color or who wear clothes that cover the whole body such as in the Middle East (54).

Furthermore, maternal vitamin D status can have a direct effect on fetal and infant immune programing. To date, multiple studies have reported that maternal vitamin D insufficiency and deficiency can also lead to child allergies (55, 56), eczema, asthma (57), and autoimmune diseases (58–60). Maternal serum 25(OH)D_3_ levels can directly affect infant vitamin D levels and immune programing (54, 61), and interestingly 1,25(OH)_2_D_3_, can cross the placenta and enter the fetal cord blood (39). Maternal serum 25(OH)D_3_ levels directly correlate with concentrations in the umbilical cord at birth (62), suggesting that maternal vitamin D might influence fetal immune response and tolerance like regulatory T cells (Tregs) stimulation of the offspring (32). D_3_ can also block lipopolysaccharides (LPS)-induced translocation of nuclear factor kappa light chain enhancer of B cells (NF-kB) p65 from the cytoplasm to the nuclei in placental cells, which prevents the activation of downstream target inflammatory genes (63).

Supplementing mothers with vitamin D have proven to be an effective method to ensure infant vitamin D sufficiency. Disanto et al. (58) performed a study in women from the UK and concluded that gestational UVB exposure could affect whether or not their offspring would suffer an immune-related disease such as colitis and MS due to vitamin D_3_ deficiency. In another study, mothers who were supplemented with 6,400 IU/day of vitamin D were able to effectively and safely provide their infant with adequate D_3_ through just breastfeeding (64). Interestingly, vitamin D supplementation induced the antimicrobial peptide (AMP), cathelicidin, which protected both mother and fetus from *Staphylococcus epidermidis* infections, which is a major cause of preterm sepsis (65).

Maternal vitamin D status is an important health concern that needs more attention especially since supplementation in the mother and/or infant has proven to be effective at improving serum 25(OH)D_3_ levels (56). For this reason, it is recommended that women who are pregnant or breastfeeding supplement daily with vitamin D in order to meet their daily recommended intake requirements to prevent deficiency and possibly avoid adverse pregnancy, birth, and offspring immune outcomes (31, 66). Some study outcomes have been inconclusive on the role of maternal vitamin D supplementation on the offspring’s immune programing [(53, 67); Figure 2].

Other recent studies in humans have demonstrated that 1,25(OH)_2_D_3_ may directly interact with the gut microbiota and ameliorate dysbiosis in autoimmune patients (68). In a cohort of 3,188 IBD patients, higher plasma 25(OH)D_3_ (27.1 ng/mL) was associated with significantly reduced risk of *Clostridium difficile* infection (68). Another study in MS patients showed that supplementing with 5,000 IU of vitamin D per day for 90 days increased the abundance of *Akkermansia*, which promotes immune tolerance, as well as *Faecalibacterium* and *Coprococcus*, which both produce butyrate, an anti-inflammatory SCFA (69). A case controlled study of 7 relapsing-remitting MS patients showed that vitamin D3 treatment caused changes in *Firmicutes, Actinobacteria*, and *Proteobacteria* levels in MS patients as well as an increase in *Enterobacteria* in healthy patients and MS patients compared to those who were not treated daily with D_3_ (70).

In animal models, a cross talk between vitamin D and the gut microbiota has also been proven. C57BL/6 mice raised on a vitamin D sufficient diet had 50 times more colonic bacteria and microbial diversity than mice raised on a vitamin D poor diet (71). In the same line, C57BL/6 mice that were raised from weaning on vitamin D deficient diets presented deregulated colonic containment of enteric bacteria, which the authors believe could be a possible mechanism behind colitis susceptibility (71). In addition, vitamin D deficient mice infected with *Citrobacter rodentium* demonstrated an altered fecal microbiome composition and increased colonic hyperplasia and intestinal barrier permeability (72). In another experiment, Cyp KO mice that could not produce 1,25(OH)_2_D_3_ and received 1.25 μg/100g of food had reduced dextran sulfate sodium (DSS)-induced colitis severity and decreased *Helicobacteraceae* abundance (73). Another study in naked mole rats (*Heterocephalus gluber*) that habitually live underground and thus receive little if any UVB exposure, were administered with 25 ng of vitamin D_2_/g food every 3 days. The authors reported that rats had a 1.4-fold increase in cecal mass and in SCFA production per gram of dry matter compared to control animals, suggesting that vitamin D can modify the gut microbiota and its byproducts leading to a healthier composition (74).

All these major discoveries make a compelling argument that vitamin D can alter the gut microbiota composition and function toward a more homeostatic state. Despite these findings, it is important to note that role of vitamin D and microbiota composition in autoimmune diseases is not as simple as just the presence or absence of a deficiency. 1,25(OH)_2_D_3_ and its metabolites are the results of many integrated enzymatic and non-enzymatic transformations with numerous intermediaries that are regulated by the host genome, epigenome, and lifestyle factors such as diet and sun and microorganisms exposure. Moreover, the activity of 1,25(OH)_2_D_3_ depends on the proper function of the VDRs, which can be regulated by gut bacteria, toxins, enteric bacteria-produced bile acids, dietary fatty acids, and epigenetic changes, which will be discussed in more detail in the next section.

### The Interaction between VDRs, Gut Microbiota, and Immune Response

#### Biological Functions of VDR

Various studies investigating the hygiene hypothesis have focused on the possible connection between vitamin D deficiency and the development of autoimmune diseases. As awareness increases about the influence vitamin D has on immune responses, attention has recently turned to how the VDR function might have a role in maintaining gut and immune homeostasis.

**Figure 3:**
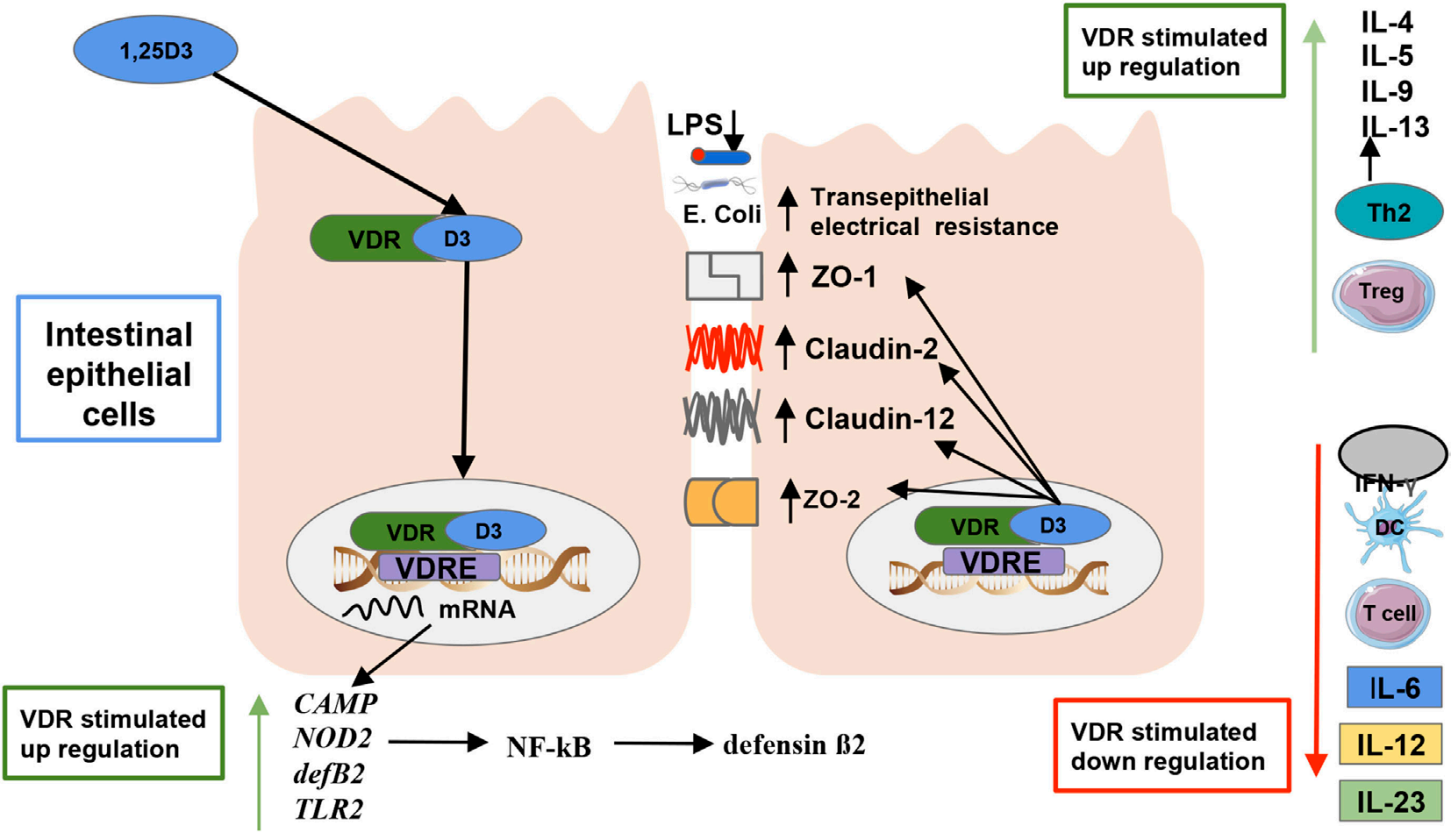
**Vitamin D’s effect on the gut microbiota and immunity**. The active form of vitamin D [1,25(OH)_2_D_3_] serves as the primary ligand for the vitamin D receptors (VDRs). The activated VDR binds to vitamin D responsive elements (VDREs) to regulate the expression of 3% of the genome (48). There are more than 1,000 genes with binding sites for VDRE, including antimicrobial peptides such as cathelicidin, β-defensin, cathelicidin antimicrobial peptide, and defensin β2 (33, 80). An activated VDR also regulates innate immune responses by controlling the genetic expression of toll-like receptor 2 (3), and nucleotide-binding oligomerization domain 2, which subsequently activates the transcription factor nuclear factor kappa light chain enhancer of activated B cells which induces defensin β2 gene expression (33). Activated VDR also plays a role in maintaining intestinal barrier function, which prevents lipopolysaccharides translocation and an ensuing inflammatory response by regulating the expression of the tight junction proteins zonulin occluden-1, zonulin occluden-2, claudin 2, and 12 in the intestine (75). VDRs can also inhibit the reduction in transepithelial electrical resistance by the pathogenic *Escherichia coli* O157:H7, which thus decreases intestinal permeability in epithelial cells (72). Furthermore, VDR promotes immune tolerance in the gut by inhibiting Th1 and Th17 cells proliferation, which produce IL-172 and IFN-γ production as dendritic cell-mediated IL-10 production, which increases regulatory T cell production promoting a T helper cell type 2 response (81, 82).

The VDR is a member of the nuclear receptor super family located in macrophages, DCs, activated T cells, and other types of cells in about 30 different tissues, including the intestines (47) and fetal tissues (75). The VDR is primarily activated by the binding of its primary ligand 1,25(OH)_2_D_3_ (76), and basically all biological actions of vitamin D are mediated by the VDR. The structure of the VDR incorporates an α-helical ligand-binding domain and a highly conserved DNA-binding domain (76). VDR, through heterodimerization with the retinoid-X receptor (RXR), then binds to vitamin D response elements (VDREs) in the regulatory region of target genes [(48); Figure 3]. VDREs are normally localized close to the promoter of genes, although evidence from recent research indicates VDR complex can operate over distances of 75 kb to regulate target gene transcription (77), increasing the potential of VDR complexes to regulate our genome (78). There are more than 1,000 genes with binding sites for VDRE, including AMPs such as cathelicidin (79), β-defensin (79), the 25-hydroxyvitamin D 24-hydroxylase (*CYP24*) gene, and cytochrome P450 family 11 subfamily A (*CYP11A1*) gene (78). In fact, ~3% of the mouse and human genomes are regulated directly or indirectly by VDRs (49), which may explain their role on preventing various diseases mechanisms (33), even in the fetal stage [(32); Figure 3].

The DNA-bound VDR/RXR heterodimers also control genetic expression via histone modification, chromatin remodeling, and RNA polymerase II binding (83). Tapp et al. (84) suggested that vitamin D status and the consequent VDR activation influences age-related cytosine–guanine dinucleotide methylation islands of human rectal mucosa in healthy subjects and thus vitamin D has epigenetic protective effects against colorectal carcinogenesis. Similarly, another study associated vitamin D deficiency and VDR activity with changes in leukocyte DNA methylation (85).

In addition to the ligand 1,25(OH)_2_D_3_, VDR gene expression may be regulated by hormones such as estrogen, thyroid hormone, and glucocorticoids, which are likely able to alter VDR mRNA/protein levels (86), but also by dietary fatty acids (87) and the gut microbiota metabolites (88, 89). Despite these findings, more investigation is required to fully understand how VDR-mediated epigenetic changes, fatty acids, hormones, and microbiota metabolites can alter immune-related genes expression in various tissues.

#### Interaction between VDRs, Immune System, and Gut Microbiota

Most of the studies in humans have reported the effects that 1,25(OH)_2_D_3_/VDR/RXR complex has on the innate immune cells (see Table 1). While investigators have made some progress there are still many unanswered questions about VDR as an immune system regulator in humans. Liu et al. (90) showed that VDR levels were greatly reduced in human with Crohn’s disease, and that VDR deletion in mice led to severe colitis.

**Table 1:** **Effects vitamin D and its biologically active form [1,25(OH)_2_D_3_] on the immune system**.

Immunological effect	Reference
**Anti-inflammatory effects**	
Promote the maturation, survival, and apoptosis of dendritic cells (DC), which is a key mechanism of the adaptive immune system	(81)
Inhibit T cell proliferation, IL-2 and IFN-γ production, and T helper 1 (Th1) cells development	(81)
Decrease B cells and antibody-secreting cells like IgG and IgM	(91)
Promote regulatory T cell (Treg) proliferation by increasing C–C motif chemokine ligand 22 expression	(47)
Regulate toll-like receptor (TLR)-2 expression	(3)
Activate macrophages, DCs, and T cells	(75)
Promote autoimmune homeostasis by stimulating Treg and inhibiting TLR8 activity	(92)
Inhibits IFN-γ and IL-17 production and induce Tregs. Tregs, in turn, induce apoptosis, inhibit Th1 and T helper 17 immune responses, and produce IL-10 and TGF-β1	(82)
Regulate the expression of mRNAs for inflammatory cytokines such as IL-1, IL-10, IL-17, and IFN-γ, as well as levels of CD3+, CD4+, CD8+, and CD19+	(71)
Downregulate pro-inflammatory cytokines such as IL-1, IL-8, IL-, IL-17, and TNF-α	(72)
Stimulate CD4(+) CD25(−) T cells and inhibit IFN-γ, IL-17, and IL-21. Work synergistically with IL-2 to produce CTLA-4 and FoxP3 Treg cells	(49)
**Antimicrobial properties**	
Upregulate the production of antimicrobial peptides such as cathelicidin and β-defensin and toll-like receptor 2	(3, 43)
Increase cathelicidin antimicrobial peptide and β-defensin expression	(33, 80)
Regulate cathelicidin which activates the transcription of autophagy-related genes *Beclin-1* and autophagy-related 5 in human monocytes	(93)
Induce nucleotide-binding oligomerization domain 2 in monocytic and epithelial cells which then stimulates NF-kB and defensin β2	(33)
Activate peroxisome proliferator activated receptor-gamma and alpha, glucocorticoids and androgens	(94)
Inhibit growth of the parasite *Toxoplasma gondii* possibly by inhibiting intra cellular proliferation *in vivo* and *in vitro*. Though the exact mechanisms are unknown	(95)
**Intestinal barrier maintenance**	
Control the expression of the tight junction proteins zonulin occluden-1, zonulin occluden-2, and can up regulate claudin 2 and 12 and down regulate cadherin-17 in the intestine	(75)
Inhibits a reduction in transepithelial electrical resistance between intestinal epithelial cells by pathogenic *Escherichia coli* O157:H7, which decreases intestinal permeability in epithelial cells	(72)
Increase transepithelial electrical resistance and decreasing LPS levels in Caco-2 cells that were either incubated or not with DSS	(96)
**Dysbiosis prevention**	
Attenuate irritable bowel disease through its anti-inflammatory properties and the prevention of dysbiosis	(82)

It is suggested that, on the one hand, VDRs negatively regulate bacteria-induced NF-kB activity in the gut (97). For example, a study in pregnant mice showed that maternal supplementation of 25 µg/kg vitamin D prior to an injection of 100 µg/kg of LPS-activated VDR signaling, which inhibited the pro-inflammatory NF-kB p65 pathway and genetic expression of the inflammatory cytokines TNF-α, IL-Iβ, and IL-6 (98). On the other hand, VDRs may contribute to maintenance of intestinal barrier function by preventing increased intestinal permeability, dysbiosis, inflammation, and a lack of immune tolerance in the gut (82). VDRs have been shown to regulate the expression of the tight junction proteins zonulin occluden-1, zonulin occluden-2 through the up regulation of claudin 2 and 12 and downregulation of adherin-17 in the intestine. All of these proteins are essential for maintaining intestinal barrier function and thus immune homeostasis and the prevention of autoimmune diseases like IBD (75). In accordance, VDR KO mice have shown to be more susceptible to LPS-induced endotoxemia, have higher expressions of inflammatory cytokines (e.g., TNF-α, IL-1a, IL-1β, IL-10, IL-21, and IFN-γ), and experience more weight loss, bleeding, ulceration, septic shock, and death compared to wild-type mice (80). Additionally, Zhao et al. (96) discovered that VDR expression increased transepithelial electrical resistance between the tight junctions and decreased LPS levels in Caco-2 cells that were both incubated or not with DSS leading to less intestinal permeability. Similarly, VDRs can also inhibit the reduction in transepithelial electrical resistance by the pathogenic *Escherichia coli* O157:H7, which thus decreases intestinal permeability in epithelial cells (72).

Additionally, Cantorna et al. (82) suggested that VDRs might regulate the gastrointestinal microbiota composition and prevent increased pathogenic proliferation in the gut by inhibiting Th1 and Th17 cells (which produce IL-17 and IFN-γ) and inducing Tregs and AMPs. To further confirm the association between VDR and microbiota composition, Wang et al. (99) analyzed gut microbiota data from a published VDR KO mouse model, confirming that the loss of VDR activity in mice substantially affects the Bray-Curtis beta diversity index in the gut (a measure of inter-individual microbiome variability). In humans, the same authors reported that VDR consistently influences individual bacteria taxa, such as *Parabacteroides* (99).

Animal models have been more promising showing the potential role of VDRs has on autoimmune diseases and microbiota composition inhibiting pathogenic proliferation in experimental murine colitis models. Wu et al. (89) discovered that VDR KO mice presented higher levels of enteric *Salmonella typhimurium*, increased pro-inflammatory NF-kB activity and higher levels of mortality upon infection compared to the control group. Additionally, they observed that mice infected with *Salmonella* presented increased VDR protein activity in the upper and lower intestinal epithelial crypts independent of its ligand 1,25(OH)_2_D_3_. Similarly, Wu et al. (97) reported that VDR KO mice had a down regulation at the transcription and translation level of the autophagy-related 16 like 1 (ATG16L1) gene, which resulted in impaired Paneth cell function, dysbiosis, and inflammation. Autophagy is a highly conserved process that is involved in intracellular homeostasis through the degradation and recycling of cytosolic contents and organelles, as well as in promoting the removal of intracellular microbes and immunity against infection (97). Remarkably, the same authors demonstrated that the absence of intestinal epithelial VDR increased susceptibility to DSS-induced colitis, while decreasing butyrate-producing bacteria, *Butyrivibrio* (97).

Other mice studies have shown the negative effects VDR deletion can have on gut homeostasis. Chen et al. (100) showed that VDR KO mice were more susceptible to the pathogen *C. rodentium*, whereas another group showed that VDR KO mice presented lower levels of *Lactobacillus*, increased levels of *Clostridium* and *Bacteroides*, as well as higher risk for infections, cancer, inflammation and other conditions compared with wild-type mice (101). Similarly, VDR KO mice had more bacteria from the Bacteroidetes and Proteobacteria phyla and fewer bacteria from the Firmicutes and Deferribacteres phyla in the feces compared to wild type (73). Recent evidence has shown that certain pathogenic microorganisms such as *Salmonella typhimurium, Borrelia burgdorferi, Cytomegalovirus, Mycobacterium leprae, Aspergillus fumigatus*, and *Mycobacterium tuberculosis*, the Epstein–Barr virus (3, 89, 102), and HIV (103) block or downregulate VDRs.

Interestingly, the gut microbiota has the capacity to produce secondary bile acids [e.g., lithocholic acid (LCA), glycine-conjugated LCA, and 3-keto-LCA from 7α-dehydroxylated primary tauro-chenodeoxycholic acid] distinct from the liver that have the potential to bind to VDR (104). A very recent study by Wang et al. (99) revealed through a genomic analysis in humans that *Parabacteroides* contained pathways involved in secondary bile acid metabolism and could thus indeed be associated with bile acid production. The interplay between VDR and *Parabacteroides* involved two genes associated with bile acid metabolism, the cytochrome P450 family 27 subfamily member 1 (CYP27A1), and the nuclear receptor subfamily 5 group A member 2 (NR5A2). Additionally, they found a positive correlation between *Parabacteroides* abundance and LCA concentrations in serum (99). The possibility that VDR acts as a key mediator in the gut–liver signaling axis and microbiota metabolism in humans motivates substantial new research directions (99).

Western lifestyle factors that affect the microbiome, such as the Western diet, antibiotics, toxins, and probiotics, could have the potential to affect the production of microbiota’s secondary bile acids, which in turn modify the function of VDR. Ongoing research is seeking to build models that predict the pattern of bile acids when certain microbiome members and metabolites are present in the gut, and the physiological effects they have on host VDRs and other transcription factors (104). Therefore, it is necessary to expand our knowledge about how VDRs relate to the human microbiome and whether those associations could be restricted to specific bacterial genera or species.

Altogether, these results illustrates that VDRs play a potentially crucial role in controlling gut homeostasis and attenuating autoimmune symptoms that require further experiments in humans. For these reasons, Waterhouse et al. (102) believe that restoring VDR function, not just serum vitamin D levels, is key to preventing or improving autoimmune symptoms. Murine model experiments clearly demonstrate that VDR deletions might exaggerate colitis demonstrating that VDR acts as a master regulator of intestinal homeostasis and establishes a unifying link between VDR, autophagy, maintenance of intestinal barrier, the production of AMPs, the intestinal microbiota, and innate immunity, all factors that have been implicated in the pathogenesis of autoimmune diseases.

#### VDR Polymorphisms and Improper VDR Function

Given the potential role VDRs have on immune responses and intestinal homeostasis, VDR genetic variants have also been studied as potential factor of autoimmune diseases since they may influence VDR activity. VDR is encoded by a large gene (>100 kb) mapped to chromosome 12q12-14. Its 14 exons spanning ~75 kb (105) exhibit a high number of polymorphisms, with at least 4,710 reported variants, most of which are either undetectable or occur at a low frequency in the general population and appear to have no potential functional significance according to the dbSNP database 2016. Among the known VDR polymorphisms, the most common single-nucleotide polymorphisms (SNPs) that influence VDR expression within the immune system include *Bmsl* (rs1544410), *Apa*I (rs7975232), *Taq*I (rs731236), and *Fok*I (rs10735810) (106). *Bsm*I, *Apa*I, and *Taq*I have been shown to be in strong linkage disequilibrium (LD) (107). Although their functional significances remain unknown, LD in combination with one or more functional polymorphisms elsewhere in the VDR gene are believed to explain observed associations between the VDR gene and autoimmune diseases (108).

A case-controlled study including 160 patients with MS and 150 healthy controls revealed the protective role of TT genotype of *Taq*I (ORs| < |1), CC genotype of *Apal*, and GG genotype of *Bsm*I (ORs| < |1), suggesting that VDR polymorphisms seem to have a notable connection with MS pathogenesis; however, studies in big population that analyze the functional work on the gene structure and its function are needed (109). Another study in 158 European Caucasians with ulcerative colitis, 245 with Crohn’s disease and 164 cadaveric renal allograft donor controls demonstrated that there were significantly more people who were homozygous for the *Taq*I polymorphism at codon 352 of exon 8 (genotype tt) among patients with Crohn’s disease (frequency 0.22) than patients with ulcerative colitis (0.12) or controls (0.12) (110).

Recently, Wang et al. (99) found that variants in the VDR gene were among the 42 significant loci and accounted for 0.75% of the microbiota variation in a cohort of 1,812 northern-Germans. They have shown for the first time that genetic variation at the VDR locus significantly impacts microbiota composition in the gut, although large sample sizes and adequate statistical power are needed in future assessments.

The absence of studies with large cohorts in this field does not mean that VDR SNPs have no biological function or that all positive associations are a case of false causation. It does show, however, that discovering the underlying biological functions of these SNPs, if they exist, will not be an easy task. Future case–control studies will have to be carefully conducted and have sufficient power in order to detect associations much weaker than those currently postulated.

The increasing interest in the epigenetic control of VDR regulation and possible significance for diseases (86) has shown that the VDR promoter region can be methylated which may affect its function. The promoter region of the VDR gene lies in a GpC-rich island and contains strong regulatory elements for its transcriptional activity (111). Disruption of promoter activity by DNA methylation is an epigenetic inactivating mechanism frequently observed in tumor-suppressor genes (112). Genetic variants of these sites might affect the methylation boundaries of the promoters, which could be the most under-explored aspect of VDR gene regulation and its role in health and disease (113). More studies are needed to fully understand the role VDR function and VDR genetic and epigenetic modifications play in preventing autoimmune diseases and dysbiosis.

## Conclusion

Several studies have positively correlated vitamin D deficiency with autoimmune diseases. Promising studies in humans have demonstrated that vitamin D_3_ supplementation can lead to an increase in beneficial bacteria, such as *Ruminococcaceae, Akkermansia, Faecalibacterium*, and *Coprococcus*, which can attenuate autoimmune responses. In mice, vitamin D_3_ has also been shown to modulate the gut microbiota toward a healthier composition by inducing AMPs such as angiogenin-4 and E-cadherin as well as autophagy in colitis models. The VDR is mainly activated by the binding of 1,25(OH)_2_D_3_, and the VDR complex regulates ~3% of the human genome. Studies in VDR KO mice have demonstrated that intestinal VDRs play an important role in regulating intestinal inflammation, autophagy, the production of AMPs, and the susceptibility to pathogenic infection. Furthermore, evidence is emerging that VDR is a key component in maintaining gut intestinal barrier function and preventing dysbiosis, which can attenuate inflammation. Recent studies suggest that VDR is regulated not only by vitamin D but also by enteric bacteria and other hormonal compounds, including the secondary bile acids produced by gut bacteria. The complex regulatory network that controls VDR activity including genetic and epigenetic modifications in its promoter region and how that affects the immune system remains largely unknown. Insights gained from understanding how the VDR pathway is involved in regulating the immune system and changing microbiome diversity may serve as a paradigm for understanding the rise in autoimmune diseases.

## Author Contributions

AC wrote and designed the main text and designed all figures. NM provided feedback and revision of manuscript. Both authors have edited and approved the final version of the manuscript.

## Conflict of Interest Statement

The authors declare that the research was conducted in the absence of any commercial or financial relationships that could be construed as a potential conflict of interest.

## Abbreviations

AMPs, antimicrobial peptides; ATG5, autophagy-related 5; ATG16L1, autophagy-related 16 like 1; BPA, bisphenol A; CAMP, cathelicidin antimicrobial peptide; CCL22, C–C motif chemokine ligand 22; Cyp24, 25-hydroxyvitamin D 24-hydroxylase; CYP27A1, cytochrome P450 family 27 subfamily member 1; CYP27B1, cytochrome P450 family 27 subfamily B member 1; D_3_, 1,25-dihydroxyvitamin D_3_; DCs, dendritic cells; DSS, dextran sulfate sodium; GALT, gut-associated lymphoid tissue; INF-γ, interferon gamma; IL, interleukin; IBD, irritable bowel disease; KO, knockout; LD, linkage disequilibrium; LPS, lipopolysaccharide; MS, multiple sclerosis; NF-kB, nuclear factor kappa light chain enhancer of B cells; Nod2, nucleotide-binding oligomerization domain 2; NR5A2, nuclear receptor subfamily 5 group A member 2; PPAR, peroxisome proliferator activated receptor; PRRs, pattern recognition receptors; SCFA, short chain fatty acids; SNP, single-nucleotide polymorphism; Th1, T helper 1; Th2, T helper 2; Th17, T helper 17; TLR, toll-like receptor; TNF-α, tumor necrosis factor alpha; Treg, regulatory T cell; UVB, ultraviolet B rays; VDR, vitamin D receptor; VDREs, vitamin D responsive elements; D_3_, 1,25(OH)_2_D_3_; ZO-1, zonulin occluden-1; ZO-2, zonulin occluden-2.
